# Comparison of Machine Learning Algorithms for Predictive Modeling of Beef Attributes Using Rapid Evaporative Ionization Mass Spectrometry (REIMS) Data

**DOI:** 10.1038/s41598-019-40927-6

**Published:** 2019-04-05

**Authors:** Devin A. Gredell, Amelia R. Schroeder, Keith E. Belk, Corey D. Broeckling, Adam L. Heuberger, Soo-Young Kim, D. Andy King, Steven D. Shackelford, Julia L. Sharp, Tommy L. Wheeler, Dale R. Woerner, Jessica E. Prenni

**Affiliations:** 1 Center for Meat Safety and Quality, Department of Animal Sciences, Colorado State University, Fort Collins, CO 80523 USA; 2 Department of Mathematics and Statistics, East Tennessee State University, Johnson City, TN 37614 USA; 3 Proteomics and Metabolomics Facility, Colorado State University, Fort Collins, CO 80523 USA; 4 Department of Horticulture and Landscape Architecture, Colorado State University, Fort Collins, CO 80523 USA; 5 Department of Statistics, Colorado State University, Fort Collins, CO 80523 USA; 6 USDA-ARS U.S. Meat Animal Research Center, Clay Center, NE 68933 USA

## Abstract

Ambient mass spectrometry is an analytical approach that enables ionization of molecules under open-air conditions with no sample preparation and very fast sampling times. Rapid evaporative ionization mass spectrometry (REIMS) is a relatively new type of ambient mass spectrometry that has demonstrated applications in both human health and food science. Here, we present an evaluation of REIMS as a tool to generate molecular scale information as an objective measure for the assessment of beef quality attributes. Eight different machine learning algorithms were compared to generate predictive models using REIMS data to classify beef quality attributes based on the United States Department of Agriculture (USDA) quality grade, production background, breed type and muscle tenderness. The results revealed that the optimal machine learning algorithm, as assessed by predictive accuracy, was different depending on the classification problem, suggesting that a “one size fits all” approach to developing predictive models from REIMS data is not appropriate. The highest performing models for each classification achieved prediction accuracies between 81.5–99%, indicating the potential of the approach to complement current methods for classifying quality attributes in beef.

## Introduction

In food science, chemical screening of ingredients and finished products is critical to ensure quality for food producers and consumers. Mass spectrometry (MS) is an important chemical detection platform for food analysis; however, methods typically require lengthy and complex sample preparation steps and analysis times^1^. Ambient mass spectrometry is a relatively new approach that enables ionization of molecules under ambient conditions with no sample preparation and very fast sampling times. Takats *et al*. reported the first ambient ionization approach, desorption electrospray ionization (DESI), in 2004^2^. There are now over thirty reported ambient ionization techniques spanning application areas from pharmaceutical analysis to biological imaging to forensics and explosives detection^3^. The application of ambient ionization technology for the analysis of food and in particular for the detection of food fraud was recently reviewed by Black *et al*.^1^.

Rapid Evaporative Ionization Mass Spectrometry (REIMS) is an emerging ambient ionization technique that has demonstrated applications in both human medicine and food science^4^. For example, REIMS-based tissue analysis can be used for intraoperative analysis of histological tissue for identification of cancerous tissue margins and other prognostic and diagnostic applications^5–9^. While the REIMS technology was initially developed with biomedical applications in mind, it has also proven to be a valuable tool for the analysis of food. Recently, utilization of REIMS for the analysis of meat products has generated very promising results across various classification scenarios reflective of important quality attributes such as genetic differences, production background, and sensory attributes. Balog *et al*. (2016) used REIMS to differentiate between various mammalian meat species and beef breeds with 100% and 97% accuracy, respectively^10^. Similarly, Black *et al*. used REIMS to accurately (98.9%) classify several fish species as an approach for detecting food fraud in the seafood industry^11^. Guitton *et al*. (2018) utilized REIMS to detect lipid changes in porcine muscle tissue reflective of treatment with ractopamine, a common growth-promotant used to increase muscle mass in swine^12^. Verplanken *et al*. (2017) successfully segregated pork carcasses with and without boar taint, an important sensory attribute related to pork quality^13^. Importantly, these examples illustrate the potential value of using REIMS to generate molecular scale information as an objective measure for the assessment of meat quality.

To utilize molecular profiles generated by REIMS or any of the ambient ionization techniques as a means to classify samples, one must employ machine learning algorithms to generate a predictive model. Machine learning is the process of rapidly finding and characterizing patterns in complex data^14^. There are many different types of machine learning including, for example, decision tree learning, network analysis, linear regression, support vector machines, and similarity functions^14^. Each of these algorithms are based on different mathematical approaches and thus, it is expected that with variation in the types of samples, data, and phenotypes in an experiment, one algorithm can greatly outperform others in terms of prediction accuracy. The majority of REIMS applications have used linear discriminant analysis on principal component analysis (PCA-LDA) reduced data for the generation of predictive models. The PCA-LDA method performs well for classification of groups that tend to show large differences in the molecular profile of samples, such as the REIMS-meat studies described above. However, when molecular profiles of samples are not overly distinct (e.g. consumer food preference) or classification of multiple groups within a single model is desired, alternative machine learning approaches may outperform PCA-LDA^15^.

In this study, the predictive accuracy of eight different machine learning algorithms were compared for the generation of predictive models using REIMS data to classify attributes of beef based on USDA quality grade, production background, breed type and muscle tenderness.

## Methods

Institutional Animal Care and Use Committee approval was not required for this study as samples were obtained postmortem from federally inspected harvest facilities.

### Sample Collection

Beef strip loin sections (*longissimus* muscle) were collected to represent 7 carcass types [Select (n = 42), Low Choice (n = 42), Top Choice (n = 39), Prime (n = 42), Dark Cutter (n = 41), Grass-fed (n = 42), and Wagyu (n = 42)] in order to provide significant variation in beef flavor attributes, tenderness, fat percentages, and animal production. Product specifications for each carcass type were verified by Colorado State University (CSU) personnel using official USDA grades and personal communication with individual suppliers to verify origin^16^. All carcasses selected were A-maturity and had typical beef-type characteristics to avoid dairy-type and B*os indicus* breed influence. With the exception of Dark Cutter carcasses, all selected carcasses were free from dark cutting lean characteristics. Select, Low Choice, Top Choice, and Prime carcasses presented marbling scores of slight (Sl), small (Sm), modest (Mt) and moderate (Md), and slighty abundant (Slab) and moderately abundant (Mab), respectively. Additional note was taken of these 4 carcass types meeting the USDA requirements for certification of Angus influence^17^. Grass-fed carcasses were selected from cattle fed a 100% forage-based diet the entirety of their lives and carcasses had marbling scores within Sm^00^-Md^99^ (superscripts represent the numerical marbling score). Wagyu carcasses were selected from crossbred Wagyu cattle (50% Wagyu, 50% Angus) and had marbling scores within SlAb^00^-Mab^99^. Dark Cutter carcasses were selected from those exhibiting dark cutting characteristics (dark colored lean) with marbling scores from Sm^00^-Md^99^. From each half of each carcass, a 5 cm thick strip loin section was collected from a point starting at the 13^th^ rib. Each strip loin section was cut to yield a single 2.54 cm steak (NAMP 1180). The steak from the left side of the carcass was used for REIMS analysis and the steak from the right side of the carcass was used for shear force analysis. Steaks were individually vacuum packaged, aged (1 °C) for 14 d postmortem, and frozen (−20 °C) until analysis.

### Shear Force

**Figure 1:**
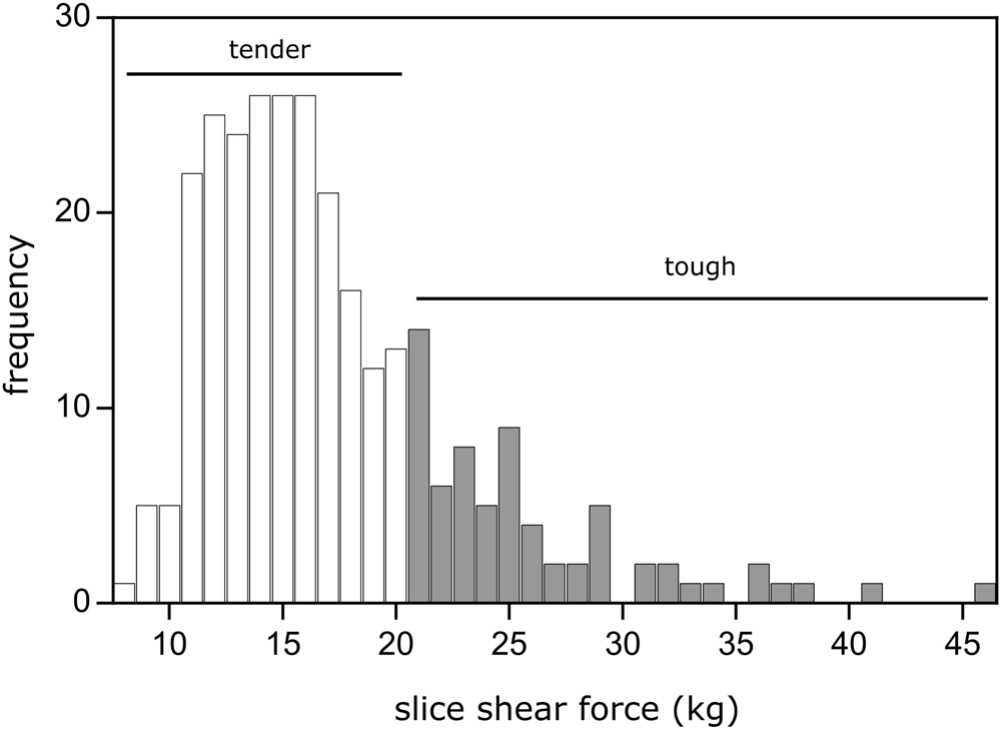
Histogram of slice shear force values and tenderness classifications as tender (<20.0 kg) and tough (≥20.0 kg).

Slice shear force (SSF) measurements were obtained from every steak using procedures described by Shackelford *et al*.^18^. Within 5 min of recording peak internal temperature, a 1 cm × 5 cm slice was removed from the steak parallel to the muscle fibers from the lateral end and sheared perpendicular to the muscle fibers, using a slice shear force machine (Model GR-152, Tallgrass Solutions, Inc., Manhattan, KS) equipped with a flat, blunt-end blade (crosshead speed: 500 mm/min, load capacity: 100 kg), resulting in a single SSF measurement for each steak. Slice shear force values are reliable objective measurements of meat tenderness and were used to divide the observations into two classifications. According to standards, steaks were classified into 2 tenderness categories, tender or tough^19^. Steaks with a SSF value less than 20.0 kg were classified as tender, and steaks with a SSF value equal to or greater than 20.0 kg were classified as tough. Measured SSF values ranged from 8.20 kg to 45.57 kg with an average of 17.33 kg and a standard deviation of 5.97 kg. The distribution of the SSF values coded by tenderness classification is illustrated in Fig. 1.

### Rapid Evaporative Ionization Mass Spectrometry (REIMS)

Chemical fingerprints of strip loin sections were acquired using rapid evaporative ionization mass spectrometry (REIMS). Prior to analysis, samples were thawed at 0–4 °C for 16–24 h. Samples were analyzed using a Synapt G2 Si Q-ToF, fitted with a REIMS ionization source coupled with a monopolar electrosurgical hand piece (“iKnife”, Waters Corporation, Milford, MA) powered by an Erbotom ICC 300 electrosurgical generator (Erbe Elektromedizin GmbH, Tubingen, Germany) using in the “dry cut” mode at a power of 40 W. A continual flow (200 μL/min) of 2 ng/mL leucine-enkephalin was introduced directly to the REIMS source during sampling. The cone voltage was set to 40 V, and the heater bias to 80 V. At least five “burns” were collected for each sample within a 2.54 × 2.54 cm square from the center of the steak (Supplementary Fig. 1), with each burn lasting approximately 1 sec. Spectra were collected in negative mode ionization from 100–1,000 m/z. Preprocessing was performed using the LiveID software (Waters Corporation) and includes lock mass correction (leucine-enkephalin), background subtraction using standard Masslynx preprocessing algorithms, and normalization to total ion current. Peak binning was performed at intervals of 0.5 m/z resulting in a total of 1,800 bins. The bins from the five burns were summed to create a single value for each sample. Mass bins in the range of 550–600 were excluded from the data matrix to remove the internal standard signal (leucine-enkephalin, m/z 554.632) resulting in a final data matrix containing 1,700 variables (m/z bins) and 290 observations (samples).

### Data Analysis

All data reduction, machine learning, and evaluation of predictive models was performed within the R statistical environment^20^.

Data were grouped together to create the desired classifications for each model set. The classification groups and four model sets are described in Table 1 and include “Main”, “Specialized”, “Breed”, and “Tenderness”. Due to similarities in sensory performance (data not shown), Select and Low Choice, as well as, Top Choice and Prime samples were combined to create two new classification groups: Low Choice/Select and Top Choice/Prime. The Main model set used the five classification categories: Low Choice/Select, Top Choice/Prime, Dark Cutter, Grass-fed, and Wagyu. The Specialized model set exclusively used the Wagyu, Grass-Fed, and Dark Cutter classifications. The Breed model set was based on classification of Angus or non-Angus as determined from the carcass meeting the USDA requirements for certification of Angus influence or not^17^. Additionally, only data from Select, Low Choice, Top Choice, and Prime carcasses were used in the Breed model set. Lastly, the Tenderness model set was defined by the slice shear force measurement as described above.

**Table 1:** Summary of classification groupings and number of observations used for each of the four model sets.

Classifications (# of observations)	Model Sets			
	Main	Specialized	Breed	Tenderness
Dark Cutter (41)	×	×		
Top Choice/Prime* (81)	×			
Low Choice/Select* (84)	×			
Wagyu (42)	×	×		
Grass fed (42)	×	×		
Tender (215)				×
Tough (74)				×
Angus (159)			×	
Not Angus (46)			×	

### Data Pre-processing With Dimension Reduction

Dimension reduction was performed using (i) principal component analysis (PCA), (ii) feature selection (FS), or (iii) PCA followed by FS (PCA-FS). PCA dimension reduction was performed using the *PCA* function in the package *FactoMineR* with unit variance scaling^21^. FS is a supervised method of data reduction where the predictors chosen are specific to the separation of the given classes. FS was performed separately for each model set in the study (i.e., Main, Specialized, Breed, and Tenderness) using the *caret* R package^22,23^. Data was pre-processed by removing highly correlated m/z bins (Pearson’s |r| > 0.90; *findCorrelation* function) followed by the *rfe* function and finally assessed with five-fold cross validation. During the feature selection process, all 1,700 m/z bins were considered to account for all possible selected variables. PCA-FS consisted of performing a similar feature selection process on the principal components, rather than the 1,700 mass bins. PCA-FS was performed using 10-fold cross validation because it was significantly more computationally efficient to conduct FS on the principal components than on the entire set of 1700 m/z bins. For all model assessment performed in this study, X-fold cross validation refers to removal of (100/X)% of the data as a validation set where the remaining (100-(100/X))% is used as the training set. This procedure was repeated X times and the average of the prediction accuracy is recorded.

### Machine Learning Algorithms To Predict Beef Quality

A total of eight machine learning algorithms were compared for predictive accuracy of each model set. These include: (1) support vector machine with a linear kernel (SVM-L), radial kernel (SVM-R), and polynomial kernel (SVM-P), (2) random forest (RF), (3) K-nearest neighbor (Knn), (4) linear discriminant analysis (LDA), (5) penalized discriminant analysis (PDA), (6) extreme gradient boosting (XGBoost), (7) logistic boosting (LogitBoost), and (8) partial least squares discriminant analysis (PLSDA). An initial screening of all the machine learning algorithms except PLSDA was performed using the *train* function in the *caret* package. PLSDA is not supported in the *train* function and thus PLSDA models were constructed using the *plsDA* function^24^ built into the *DiscriMiner* package. Visualization plots of the PLSDA model based on the PCA-FS reduced data were generated using the *ggpubr* package. Ellipses were drawn using a 90% probability region. 10-fold cross validation was used to evaluate the prediction accuracy (correct predictions/total predictions × 100) of all eight machine learning algorithms. For PLSDA, a manual 10-fold cross-fold validation was conducted using the *predict* function.

For each of the four model sets (Main, Specialized, Breed, Tenderness), the eight machine learning algorithms were applied on data following the four pre-processing options (no reduction, PCA, FS, or PCA-FS reduction). Prediction accuracies of each model were determined by 10-fold cross validation.

The top three performing models (in terms of prediction accuracy based on 10-fold cross validation) for each model set were further optimized via parameter tuning using the specific functions in R rather than the *train* function (Table 2). Following optimization, the top three models were revalidated using 100-fold cross validation for each model set (a higher fold cross validation was used to more precisely estimate the precision accuracy).

**Table 2:** R packages and functions used for the development of final predictive models.

Machine Learning Algorithm	R Function	Package
LDA	lda()	MASS
LogitBoost	LogitBoost()	*caTools*
PDA	fda ()	mda
SVM	svm()	*e1071*
XGBoost	xgboost()	*xgboost*
PLSDA	plsDA()	DiscriMiner

## Results and Discussion

REIMS analysis of the beef samples in this study resulted in a data matrix that included 1,700 m/z bins per sample, for a total of 290 samples in the full experiment. This type of unbalanced data, typical in omics experiments, (i.e. many more predictors than observations) can lead to issues when applying machine learning algorithms such as slow computational time and the possibility of model overfitting^15^. To address this challenge, three types of dimension reduction methods were compared in this study including: principal component analysis (PCA), feature selection (FS), and PCA followed by FS (PCA-FS). In PCA, the data transforms the predictor variables into a smaller number of variables (termed principal components, PCs), based on predictor co-variation (direction and magnitude) in the data, and is an unsupervised method^22^. When employed for data reduction, all of the PCs are retained and the output is a new dataset that represents 100% of the variation in the data using substantially fewer predictors. In FS, a supervised, recursive feature elimination method is used to remove predictor variables from the data matrix^22^. FS applies a backwards selection of predictors based on a ranking of predictor importance. The less important predictors are sequentially eliminated, with the goal of finding the smallest subset of predictors that can generate an accurate predictive model. The PCA-FS method is based on reducing the data into components, then performing FS on components to reduce redundancy in the data matrix.

Here, the PCA, FS-, and PCA-FS methods were applied to the data matrix of 1,700 m/z bins for each of the four model sets. The PCA, FS, and PCA-FS methods reduced the data matrix to 226 (13.3%), 240 (14.0%), and 22 (1.3%) mean predictor variables, respectively (Table 3). The number of predictors in the processed data was most variable for the FS method (coefficient of variation 92%; among the four model sets), indicating that PCA is likely to produce a more consistently sized data matrix for predictive modeling.

**Table 3:** Number (percent) of predictors for each dimension reduction technique for each model set.

Model Set	Original	PCA	FS	PCA-FS
Main	1,700 (100%)	289 (17%)	229 (13.47%)	24 (1.41%)
Specialized	1,700 (100%)	124 (7.29%)	60 (3.53%)	8 (0.47%)
Breed	1,700 (100%)	203 (11.94%)	602 (35.41%)	38 (2.24%)
Tenderness	1,700 (100%)	289 (17%)	67 (3.94%)	16 (0.94%)

### Machine learning algorithms varied in their prediction accuracy

In this study, eight machine learning algorithms were compared for the prediction of specific quality attributes in beef based on molecular profiles generated by REIMS.

#### Partial least squares discriminant analysis (PLSDA)

**Figure 2:**
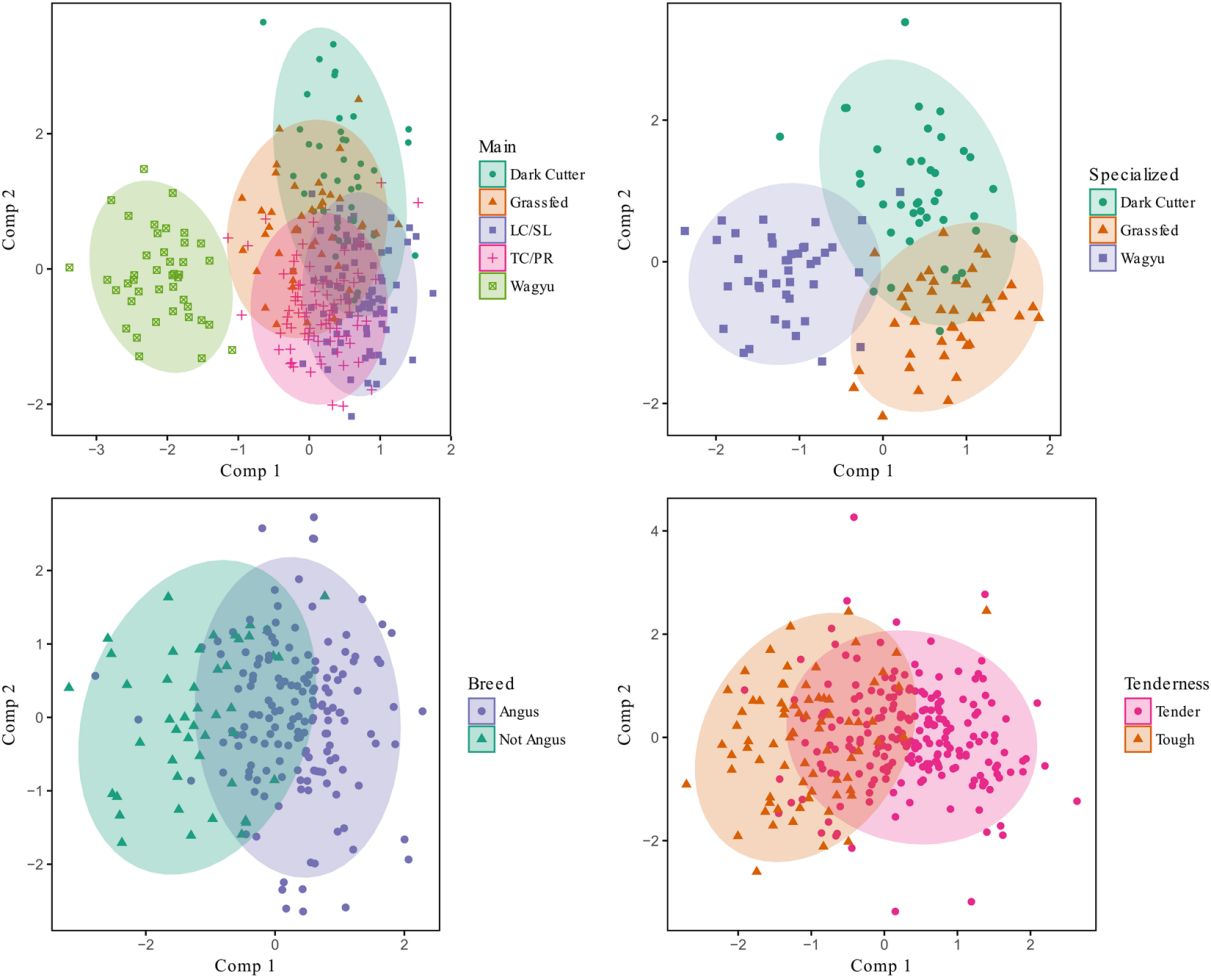
Visualization of the PLSDA model for each of the model sets. Plots represent the first two PLS components of the PCA-FS reduced data.

A model that transforms data into partial least squares components that can be then used to classify an observation. PLSDA is a common chemometrics method used in mass spectrometry ‘omics’ experiments to predict outcomes based on chemical signals^15,25^, however this algorithm can be easily misused, misinterpreted, and is prone to overfitting^25^. The PLSDA algorithm has many advantages including performance power when working with multivariate data along with methods of dealing with collinear variables^26^. PLSDA is similar to a supervised version of principal component analysis. However, in this study, the PLSDA model was used as a classification algorithm and for data visualization rather than a dimension reduction technique. The data were visualized using the first two PLS components of the PCA-FS dimensionally reduced data (Fig. 2). The Specialized model set showed clear separation between classes, with slight overlap between the dark cutter and grass-fed classes. The most overlap was observed in the Main model set where only the Wagyu class appears to separate clearly from the others. For the Breed and Tenderness model sets, there is clear separation of the classes with some overlap of the moderate values.

#### Support vector machine (SVM)

A discriminative classifier that separates clusters of observations with the use of a hyperplane. In particular, the SVM method determines the optimal hyperplane to differentiate between the classes within the data. Here, this study evaluated how the “kernel” parameter of SVM (linear, radial, or polynomial) can result in varying prediction accuracy.

#### Random Forest (RF)

A type of decision tree. Although individual classification trees tend to lack in performance compared to other machine learning algorithms, aggregating many decision trees together with methods such as bagging, random forests, and boosting can greatly increase the predictive accuracy of the model^27^. The RF algorithms can increase model performance compared to other classification tree methods by decorrelating the trees^27^. Similar to bagging, random forest methods construct *n* number of decision trees on bootstrapped training samples. However, the unique component of the random forest model is that for each decision split within a given tree, the model is only able to use *m* predictors. The parameter *m* can be set as any value less than or equal to the number of predictors (note that if *m* equals the number of original predictors it would be performing a bagging classification method). Typically, *m* is chosen to be approximately equal to the square root of the number of predictors. By only using *m* predictors at each split, the trees are decorrelated due to the same predictors not being selected for each of the trees.

#### K-nearest neighbor (Knn)

A nonparametric approach with no underlying assumption about the distribution of the data. This algorithm classifies observations based on the similarities in features between individuals. The model determines feature similarity by calculating the Euclidian distance between the features of different observations and assigns a distance value to each observations and their neighboring observations^27^. Deciding the best K value for a given data set is an optimization problem. A loop can be used to input various K values into the algorithm to find the value of K that minimizes the error rate of class prediction.

#### Linear discriminant analysis (LDA)

A parametric approach that assumes the predictors X_1_, …, X_k_ are drawn from a multivariate Gaussian distribution. LDA is a mathematically simple and robust method of classification. LDA uses linear decision boundaries for the classification of observations, and this method calculates a linear combination of predictor to separate the model’s classes^27^.

#### Penalized discriminant analysis (PDA)

An expansion of the linear discriminant analysis model. The PDA algorithm uses nonlinear spline basis functions and includes a penalty term that adds smoothness to the coefficients of the model to reduce the problem of multi-collinearity in the predictors^28^. Therefore, this penalized algorithm typically performs well when there are many highly correlated variables. When there are a large number of correlated variables within a dataset, many times, the covariance data matrix is non-invertible^27^. Including a penalization parameter in the model reduces the likelihood of singular (non-invertible) covariance matrices and results in improved classification accuracy.

#### XGBoost:

A supervised learning algorithm designed for fast computational time, especially on very large data sets. XGBoost is a form of gradient-boosted decision trees that is faster than comparable implementations of gradient boosting. Gradient boosting can generate new models based on the prediction of the residuals errors of prior models^29^. The term “gradient boosting” refers to the utilization of a gradient descent to minimize the loss when adding additional models (Brownlee, 2016).

#### LogitBoost:

A boosting logistic classification algorithm that performs as an additive logistic regression model. The Logit Boost model is similar to a generalized additive model, but rather than minimizing the exponential loss, the algorithm minimizes the logistic loss of the function. Additionally, the Logit Boost algorithm in R is trained using one node decision trees as weak learners^30^.

**Figure 3:**
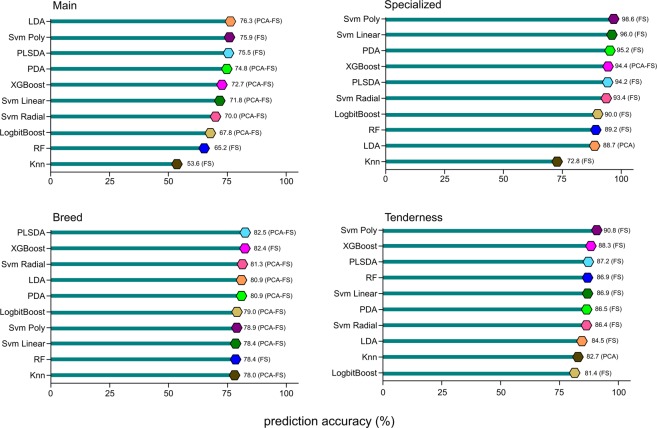
Prediction accuracies (based on 10-fold cross validation) for the top performing machine learning algorithm and data reduction approach combinations for each model set.

The performance of each machine learning algorithm and data reduction combination was assessed in the initial screening step (Supplementary Figs 1–6). Performance was evaluated in terms of prediction accuracy using a 10-fold cross validation. The best performing machine learning algorithm and data reduction combinations for each model set are summarized in Fig. 3 and Supplementary Table 1. For the two binary model sets, Breed and Tenderness, the prediction accuracies among the highest performing machine learning algorithm data reduction approach combinations to the lowest span only 4.5% and 9.4%, respectively (Breed range: 0.78–0.825; Tenderness range: 0.814–0.908). This result supports that all of the approaches generated a consistently accurate model. More variation was observed in the prediction accuracies for the complex Main and Specialized model sets with predictions accuracies spanning 22.7% and 24%, respectively (Main range: 0.536–0.763; Specialized range: 0.728–0.968).

Parameter tuning and optimization was performed for the top three machine learning algorithms for each model set. The overall highest performing machine learning algorithm and data reduction approach combination for each model set was selected based on prediction accuracy (100-fold cross validation) using the optimized algorithms. Prediction accuracies for the highest performing models are reported in Table 4. Interestingly, the most accurate machine learning algorithms were different for each model set, specifically LDA, XGBoost, and SVM (both linear and radial), although in many cases prediction accuracies of the top ranked algorithms differed by less than 1% (Fig. 3). Additionally, dimension reduction using either FS or PCA-FS was optimal for all of the top performing algorithms.

**Table 4:** Summary of final prediction accuracies based on 100 fold cross validation for the top machine learning algorithm and data reduction approach combination for each model set after parameter tuning.

Model Set	Dimension Reduction Approach	Number of predictors	Machine Learning Algorithm	Final Accuracy Rate
Main	PCA-FS	24 PCs	LDA	**81.5%**
Specialized	FS	60 mass-bins	SVM - Linear	**99%**
Breed	PCA-FS	38 PCs	SVM - Radial	**85%**
Tenderness	FS	67 mass-bins	XGBoost	**90.5%**

### Relevance of Machine Learning of REIMS data for Beef Quality Predictions

The results obtained in this study demonstrate that integrating machine learning with REIMS data can predict beef quality attributes with considerable accuracy, including quality grade, production background, breed type, and muscle tenderness. Dimension reduction improved the predictive accuracy in all cases, supporting that this is a critical step in data processing and analysis. Further, machine learning algorithms varied in their performance depending on the model set, indicating that the patterns in the chemical data (i.e. REIMS spectra) are highly complex and variable for the different facets of beef quality attributes. Thus, finding a “one size fits all” approach to generate predictive models for beef quality attributes is unlikely, and instead, evaluation of multiple algorithms should be standard practice in model development with highly complex chemical data.

Our results support the potential for REIMS analysis to be further developed to complement to the beef quality classification systems. Tenderness is a critical attribute for consumer satisfaction, which can exist independently of USDA quality grade, breed type, or production background^31–33^. Slice shear force can be used to verify guaranteed tender programs, but this method is laborious, costly, and destructive, and the industry has not widely adopted its use for individual carcass classification for product labeling^34^. Several instrument methods to classify beef tenderness have been evaluated that are less destructive than SSF and could be implemented at line speeds, but have yet to be adopted by the industry for routine use^35–40^. In this study, REIMS output, coupled with machine learning, correctly classified tough and tender carcasses with more than 90% accuracy (Table 1), indicating the potential value this approach for industry use.

Similarly, beef from Angus breed type cattle can receive significant premiums, as it is a requirement for several of the most desirable and highest quality branded beef programs. However, Angus influence is most commonly determined by visually assessing the predominance of black coloring of the live animal’s hide, rather than a true genetic test or physical documentation of lineage. Several machine learning algorithms evaluated in this study predicted Angus breed type with greater than 80% accuracy. It is important to note that this result does not represent a prediction of genuine Angus influence, but rather prediction of a carcass originating from an animal with a predominantly black colored hide. However, the results support the potential for prediction of true Angus genetic influence in future work, where carcasses with known genetic background could provide a more objective alternative to identifying Angus influence. Successful prediction of Grass-fed, Wagyu, and Dark Cutter carcasses with considerable accuracy in the current study suggest additional potential to utilize REIMS in determining and/or verifying various quality-related beef carcass characteristics.

## Conclusions

The current study demonstrates that chemical profiles generated by REIMS and interpreted with machine learning algorithms can generate predictive models for beef quality attributes such as carcass type, production background, breed type, and muscle tenderness. The optimal machine learning algorithm, as assessed by predictive accuracy, was different depending on the classification problem, suggesting that a “one size fits all” approach to developing predictive models from REIMS data is not optimal. Furthermore, in all cases, data reduction prior to modeling improved the overall model accuracy. Taken as a whole, the results presented here lay the groundwork for future evaluation of REIMS in an on-line production setting to complement current meat classification methodologies and enable objective sorting and verification of meat products by attributes with high economic value.

## Supplementary information

Supplementary Information

Supplementary Data

## Data Availability

Raw mass spectrometry data files have been uploaded to the Metabolights data repository. Pre-processed mass spectrometry data and additional metadata for each sample is tabulated in Supplementary Data Table.
