# Supplementary material for: Comparison of Machine Learning Algorithms for Predictive Modeling of Beef Attributes Using Rapid Evaporative Ionization Mass Spectrometry (REIMS) Data
Source: Sci Rep. 2019 Apr 5;9:5721. doi: 10.1038/s41598-019-40927-6 (PMC6450883; doi:10.1038/s41598-019-40927-6)
Supplement: Supplementary file 1 — Supplementary Information [file 41598_2019_40927_MOESM1_ESM.pdf]

Comparison of Machine Learning Algorithms for Predictive Modeling of Beef Attributes Using Rapid Evaporative Ionization Mass Spectrometry (REIMS) Data

Devin A. Gredell[1], Amelia R Schroeder[2], Keith E. Belk[1], Corey D. Broeckling[3], Adam L. Heuberger[4], Soo-Young Kim[5], D. Andy King[6], Steven D. Shackelford[6], Julia L. Sharp[5], Tommy L. Wheeler[6], Dale R. Woerner[1], Jessica E. Prenni[4*]

Supplementary Information

Supplementary Table 1. Prediction accuracy results for initial screening of machine learning algorithms and data reduction combinations. Values represent the top performing combination for each model set. The overall top performer for each model set is highlighted in gray.

Machine Learning Algorithm	Main	Specialized	Breed	Tenderness
Knn	0.5357 (FS)	0.7284 (FS)	0.7798 (PCA-FS)	0.8277 (FS)
LDA	***0.7632 (PCA-FS)	0.8865 (PCA-FS)	0.8089 (PCA-FS)	0.8448 (FS)
Logit Boost	0.6779 (FS)	0.8997 (FS)	0.7897 (PCA-FS)	0.8136 (FS)
PDA	0.7482 (PCA-FS)	*0.9519 (PCA)	0.8088 (PCA-FS)	0.8646 (FS)
Random Forest	0.6515 (PCA-FS)	0.8917 (FS)	0.7837 (FS)	0.8695 (FS)
SVM Poly	**0.7595 (PCA-FS)	***0.9679 (FS)	0.7892 (FS)	***0.9075 (FS)
SVM Radial	0.6996 (PCA-FS)	0.9358 (FS)	*0.8132 (PCA-FS)	0.8644 (FS)
SVM Linear	0.7179 (FS)	**0.9596 (FS)	0.7844 (PCA-FS)	0.8693 (FS)
XGBoost	0.7271 (FS)	0.9436 (FS)	**0.8237 (PCA-FS)	**0.8833 (FS)
PLSDA	*0.7552 (FS)	0.9417 (FS)	***0.8250 (PCA-FS)	*0.8724 (FS)

*** Model with highest accuracy
** Model with second highest accuracy
* Model with third highest accuracy

Supplemental Figures

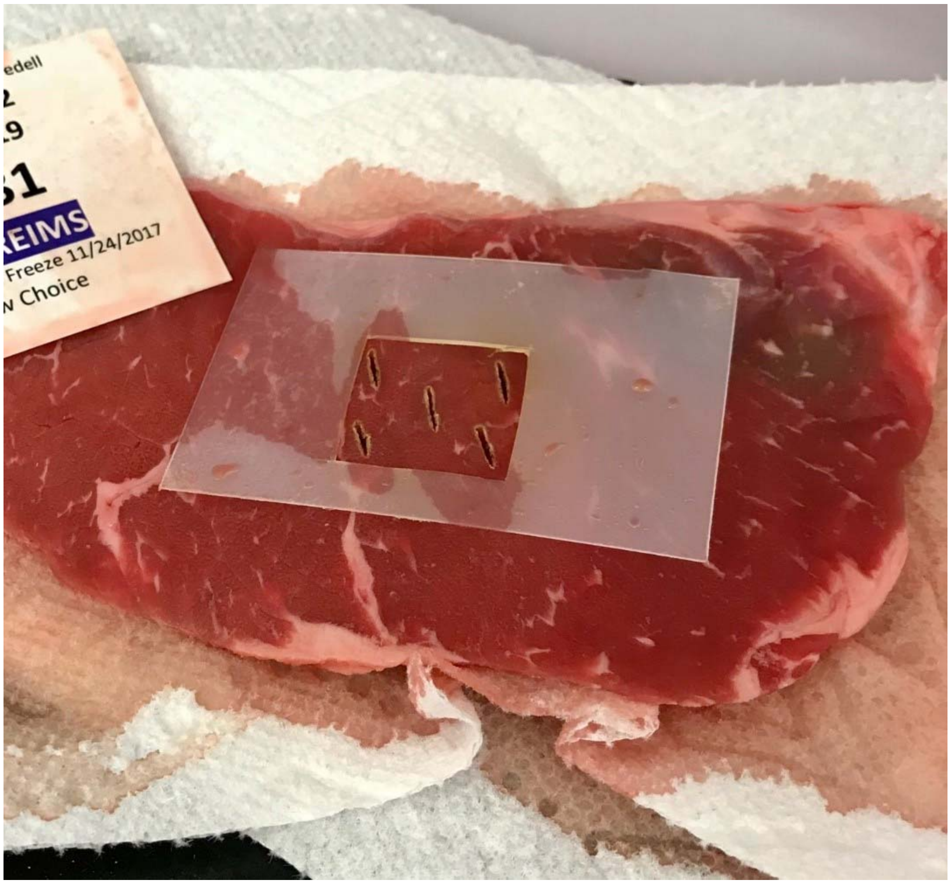

Supplementary Figure 1. Example of pattern used to collect five “burns” for each sample.

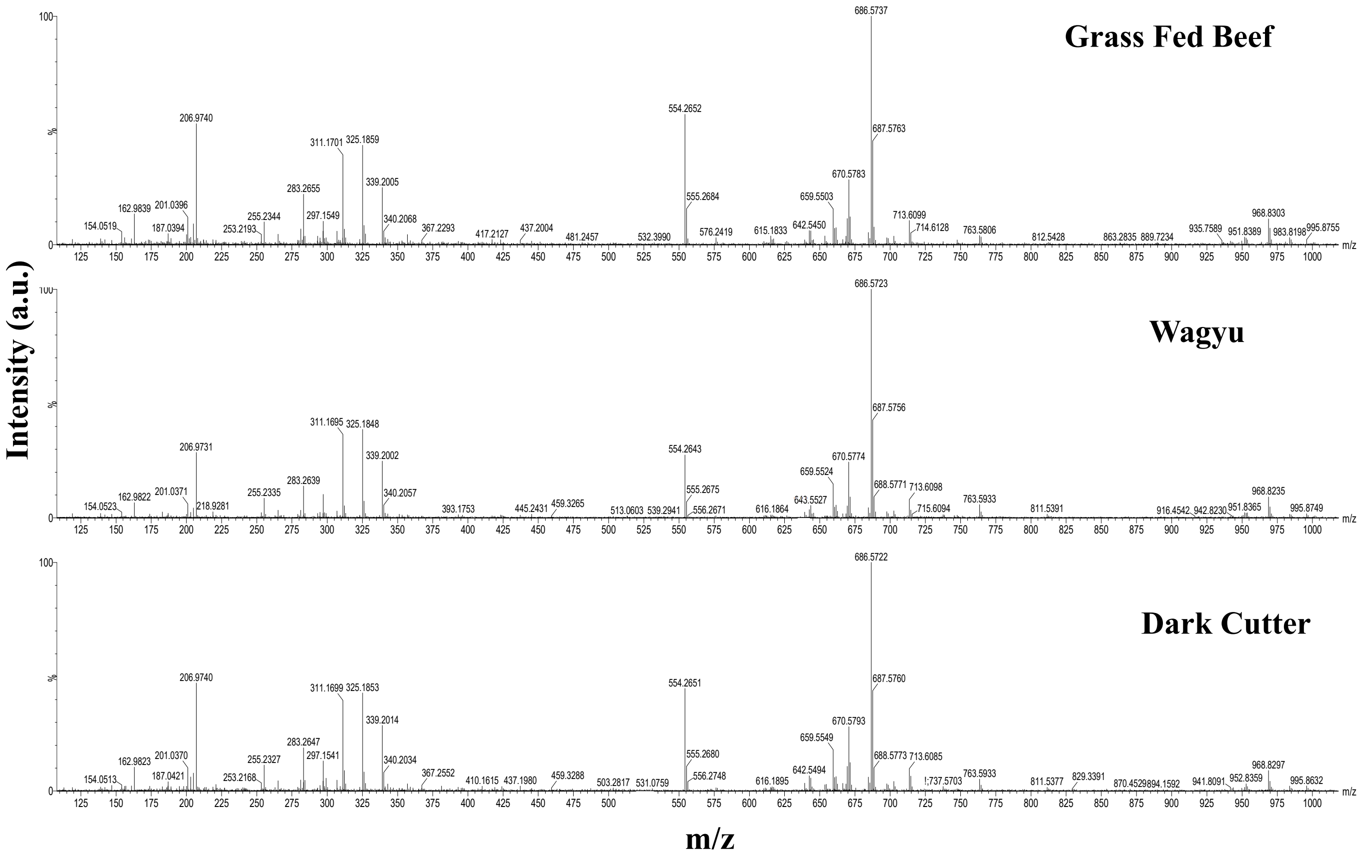

Supplemental Figure 2. Example REIMS spectra for grass fed, Wagyu, and dark cutter beef. The peak at m/z 554.26 represents the internal standard (leucine-enkephalin) which was used for lock mass correction. The peak bins (550-600) containing this signal were removed from the final data matrix before predictive modeling.

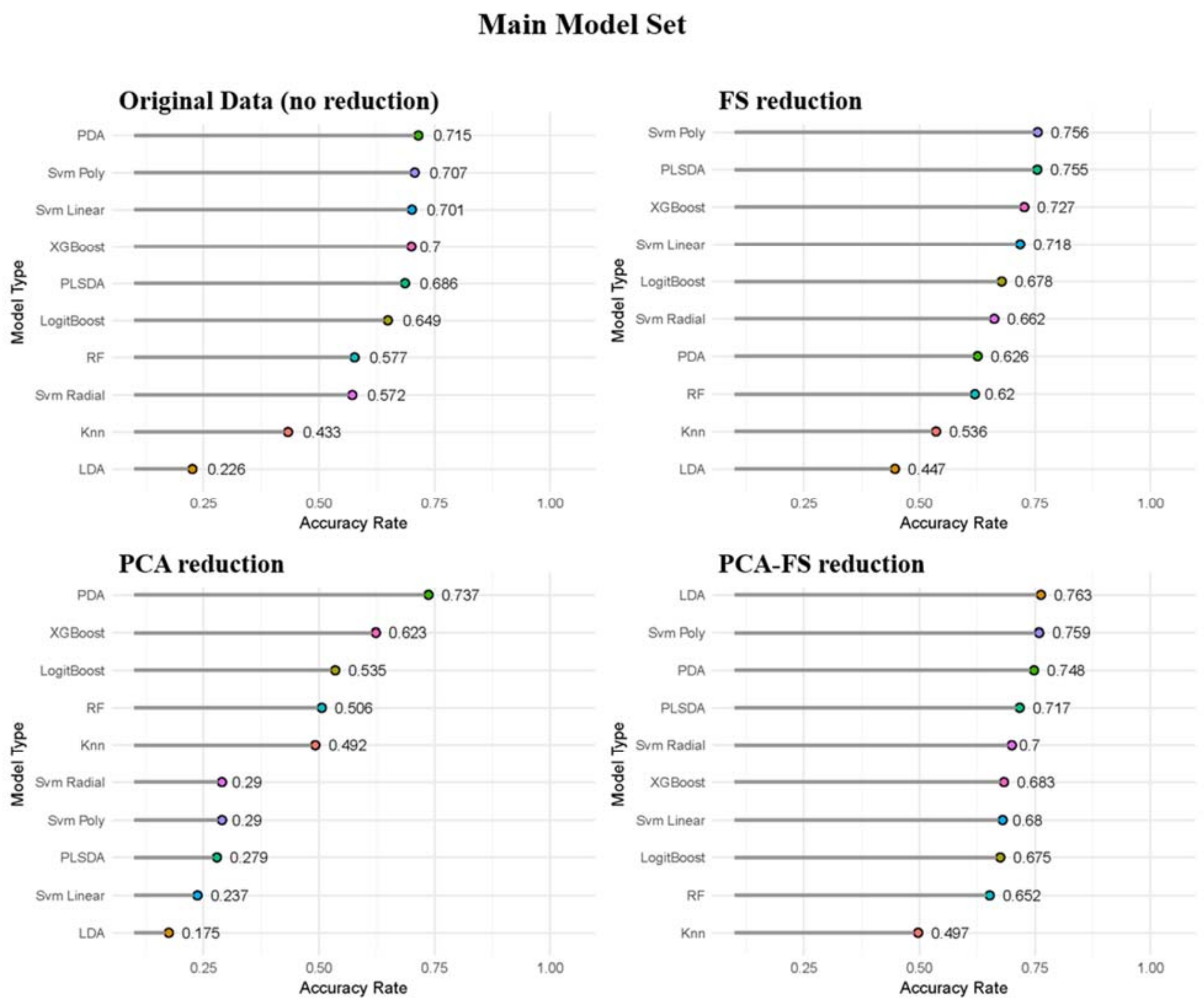

Supplemental Figure 3. Prediction accuracies (based on 10-fold cross validation) for all machine learning algorithms and data reduction approaches for the Main model set.

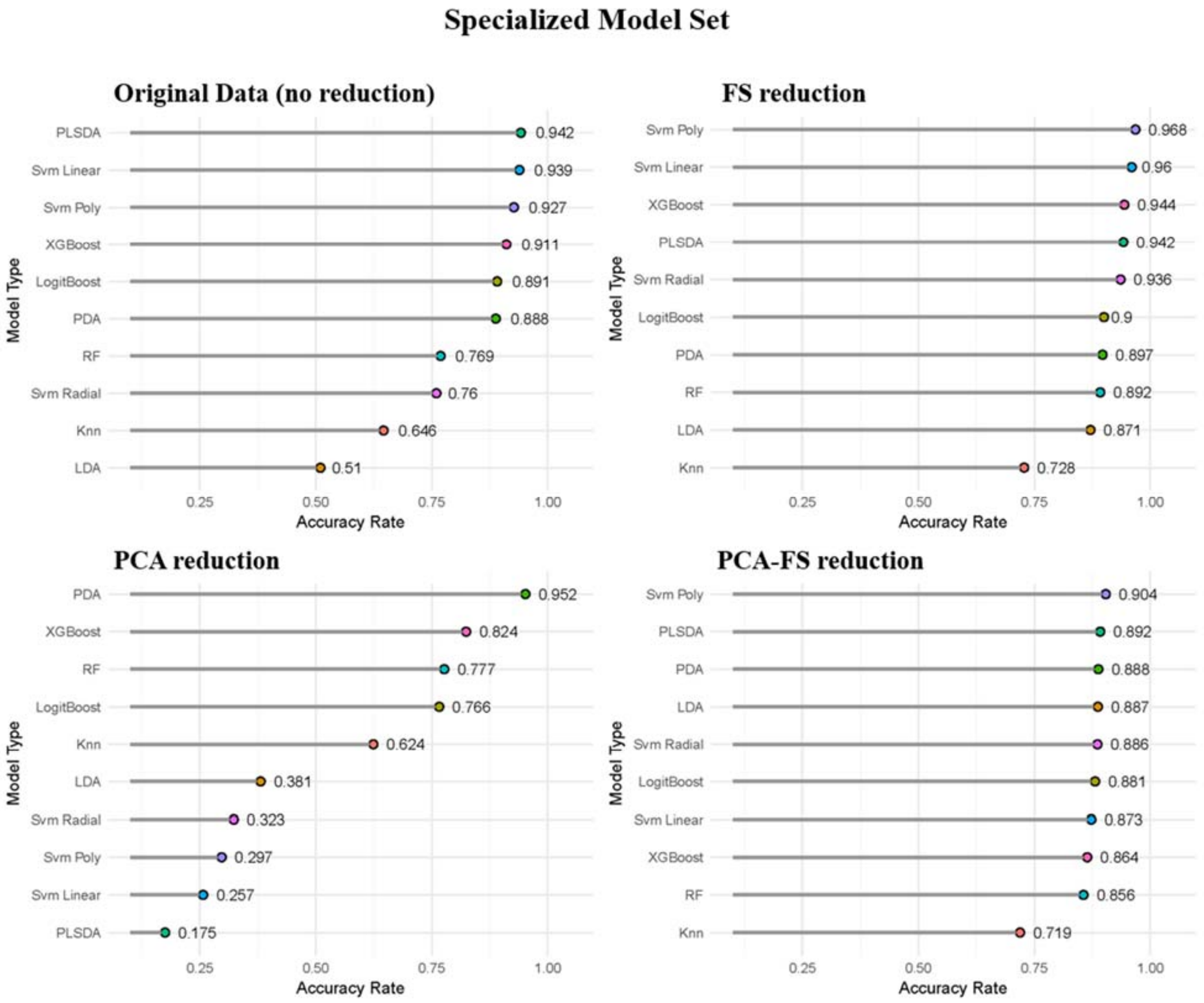

Supplemental Figure 4. Prediction accuracies (based on 10-fold cross validation) for all machine learning algorithms and data reduction approaches for the Specialized model set.

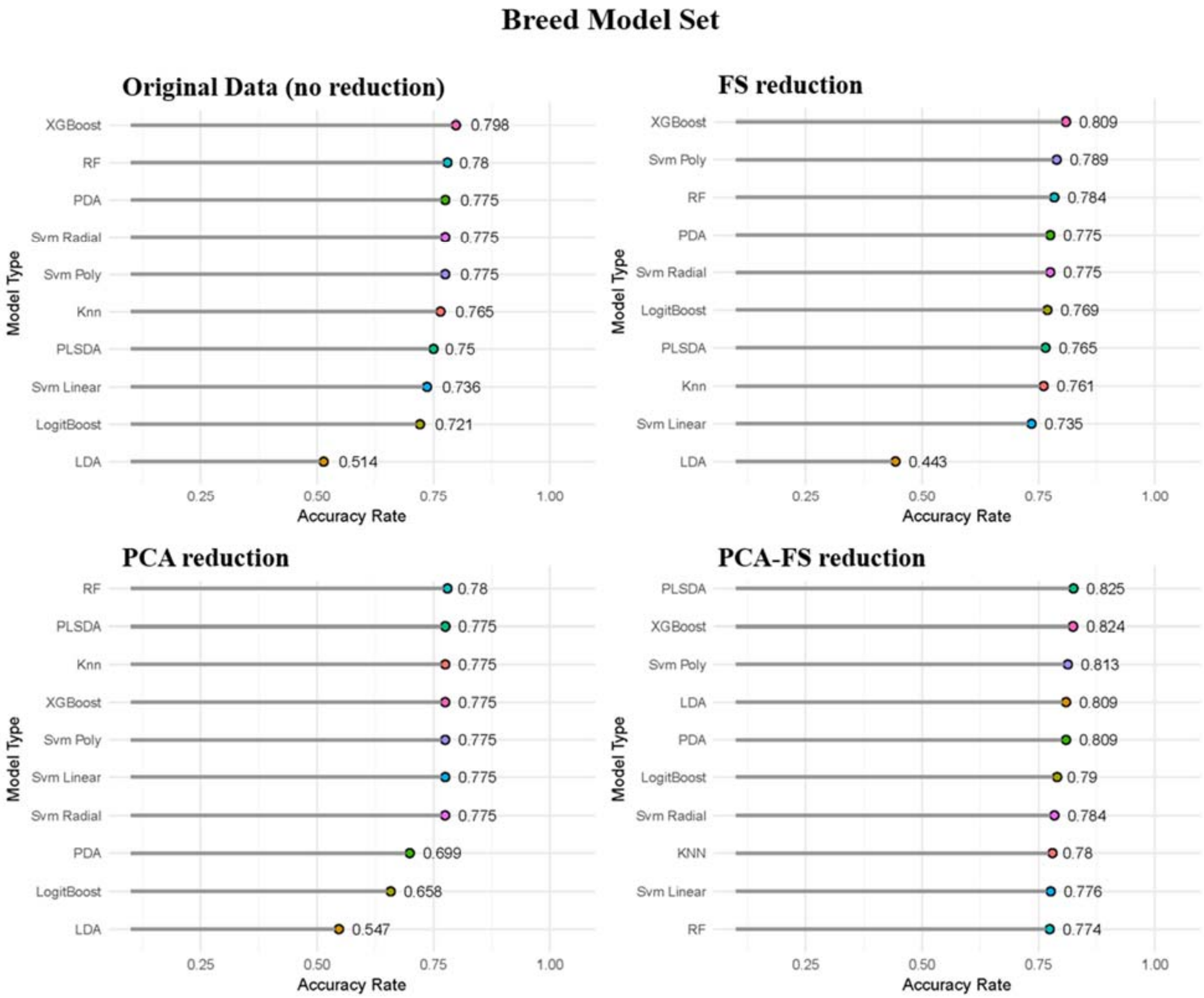

Supplemental Figure 5. Prediction accuracies (based on 10-fold cross validation) for all machine learning algorithms and data reduction approaches for the Breed model set.

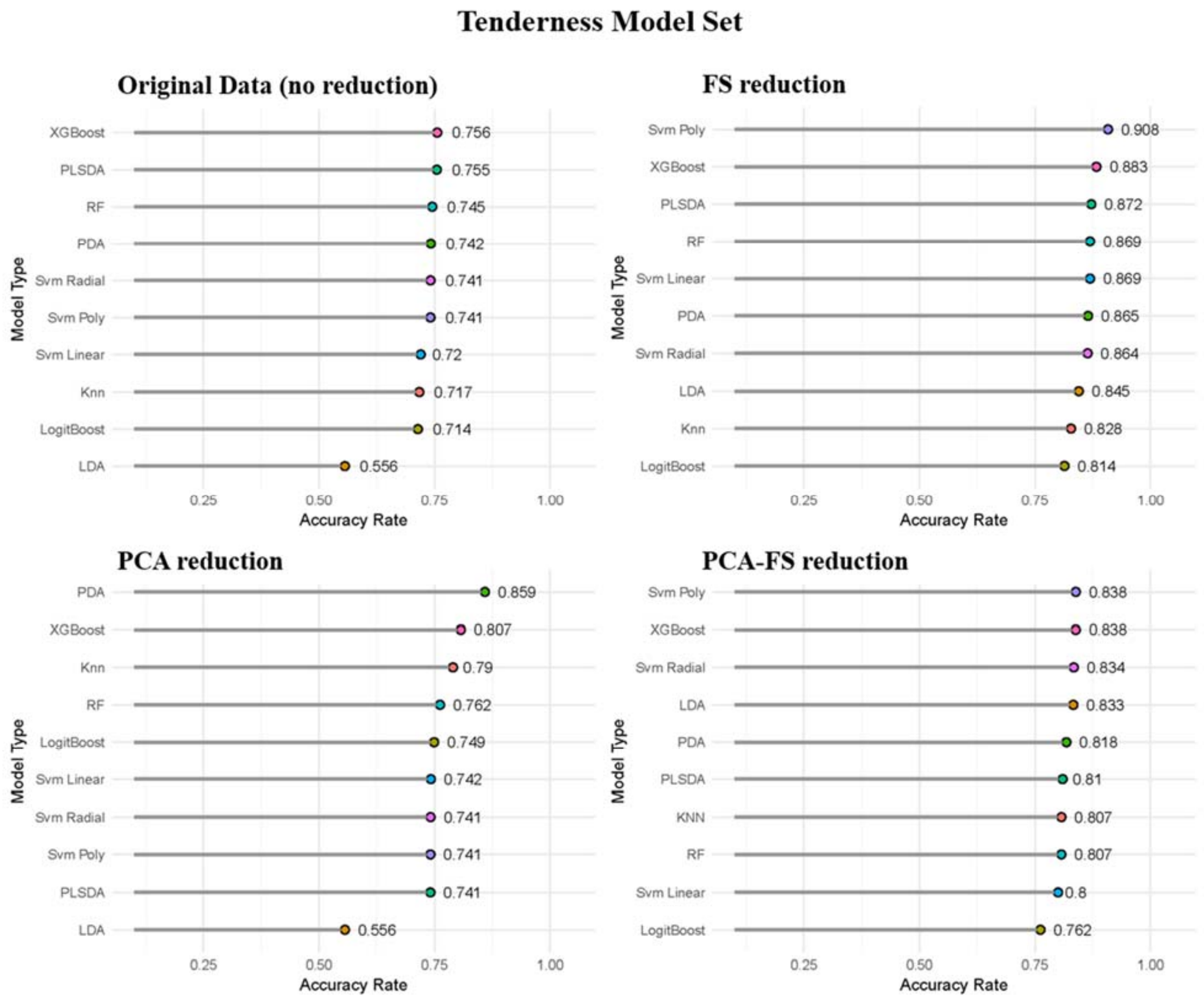

Supplemental Figure 6. Prediction accuracies (based on 10-fold cross validation) for all machine learning algorithms and data reduction approaches for the Tenderness model set.
